# Monitoring Functional Capability of Individuals with Lower Limb Amputations Using Mobile Phones

**DOI:** 10.1371/journal.pone.0065340

**Published:** 2013-06-04

**Authors:** Mark V. Albert, Cliodhna McCarthy, Juliana Valentin, Megan Herrmann, Konrad Kording, Arun Jayaraman

**Affiliations:** 1 Sensory Motor Performance Program, Rehabilitation Institute of Chicago, Chicago, Illinois, United States of America; 2 Department of Physical Medicine and Rehabilitation, Northwestern University, Chicago, Illinois, United States of America; 3 Mechanical Engineering, Massachusetts Institute of Technology, Cambridge, Massachusetts, United States of America; 4 Biomedical Engineering, Boston University, Boston, Massachusetts, United States of America; 5 Max Nader Center for Rehabilitation Technologies and Outcomes Research, Rehabilitation Institute of Chicago, Northwestern University, Chicago, Illinois, United States of America; 6 Center for Bionic Medicine, Rehabilitation Institute of Chicago, Northwestern University, Chicago, Illinois, United States of America; Semmelweis University, Hungary

## Abstract

To be effective, a prescribed prosthetic device must match the functional requirements and capabilities of each patient. These capabilities are usually assessed by a clinician and reported by the Medicare K-level designation of mobility. However, it is not clear how the K-level designation objectively relates to the use of prostheses outside of a clinical environment. Here, we quantify participant activity using mobile phones and relate activity measured during real world activity to the assigned K-levels. We observe a correlation between K-level and the proportion of moderate to high activity over the course of a week. This relationship suggests that accelerometry-based technologies such as mobile phones can be used to evaluate real world activity for mobility assessment. Quantifying everyday activity promises to improve assessment of real world prosthesis use, leading to a better matching of prostheses to individuals and enabling better evaluations of future prosthetic devices.

## Introduction

The projected number of individuals with amputations in the United States is expected to more than double in the next 40 years, primarily due to an aging population suffering from dysvascular diseases and diabetes [1]. Because 95% of all amputations caused by dysvascular diseases are lower-limb amputations, the need for lower-limb prostheses is expected to rise significantly higher. Given the majority of dysvascular amputations are in the elderly (56% over 65 years), and dysvascular conditions are often comorbid with impaired mobility prior to amputation, it is important to provide health care that is tailored to the functional capabilities of each individual.

In lower-limb prosthetics, there is a range in complexity, price, and most importantly functional tradeoffs. Lower-limb prostheses can range from a simple single-axis knee to more complicated multi-axis powered knees and ankles used to enable a more natural gait [2], [3]. A single axis mechanical knee uses mechanical friction as an adjustable brake to control the swinging of the artificial limb. Unfortunately, due to the constant friction, single-axis knees limit activities of daily living that might require variable cadence. Powered knees use advanced sensor technology including accelerometers and load sensors to provide actuation, enabling a better ability to perform most activities of daily living. Prices for prostheses can vary from the $45 Jaipur Foot, provided free of charge to beneficiaries [4] to the Power knee which can cost more than $100,000 [5]. Choosing a prosthesis that is well matched to a patient’s needs and capabilities allows more advanced devices to be allocated to the individuals that can most benefit.

There are a number of conventional methods available for assessing the ability of individuals with lower-limb amputations to undertake activities of daily living. These can be divided generally into self-report and physical measures. A number of thoroughly researched questionnaires can be used to estimate the difficulty of activities of daily living for individuals with amputations, including the Prosthesis Evaluation Questionnaire (PEQ) [6], Orthotics and Prosthetics Users’ Survey (OPUS) [7], Questionnaire for Persons with a Transfemoral Amputation (Q-TFA) [8], SIGAM mobility grades [9], Prosthetic Profile of the Amputee (PPA) [10] and the Locomotor Capabilities Index (LCI) [10]. However, self-report is inherently subjective, which can lead to increased variability and bias. Another way to infer the mobility at home is from physical measures of ambulation obtained in a clinical setting. For example, the six minute walk, the functional ambulation profile, and Timed Up and Go (TUG) can be applied to individuals wearing prostheses [11], as well as combined measures specific to individuals with amputations, such as the Amputee Mobility Predictor [12]. These self-report questionnaires and physical measures can then be used by clinicians to infer the individual’s current capabilities in daily life.

The standard system of classifying functional capability for individuals with lower-limb amputations is the Medicare Functional Classification Level, known as K-levels (Table 1) [13]. By identifying the activity level, physicians and prosthetists evaluate which prosthesis would be most beneficial. This is not simply to save money. K0 individuals may only need a prosthesis for aesthetic reasons. Lower K-level ambulators, who need more stability and only have limited community ambulation, may be adversely affected by the uncertainty inherent in powered prostheses. However, at higher K-levels advanced prostheses can have a dramatic impact on quality of life. Currently, K-levels are assigned to individuals based on the judgment of a clinician, often aided by clinical measurements at the time of assignment. Using data from everyday use of prosthetic devices promises to make this user-device matching more efficient.

**Table 1:** Medicare functional classification levels (K-levels).

K-level	Functional Description
K0	Does not have the ability or potential to ambulate or transfer safely with or without assistance
K1	Can use a prosthesis for transfers or ambulation on level surfaces at fixed cadence
K2	Can ambulate with the ability to traverse low-level environmental barriers such as curbs, stairs, or uneven surfaces
K3	Can walk with variable cadence
K4	Exceeds basic walking skills, exhibiting high impact and energy levels

One way to accurately assess mobility during daily activities is by having individuals wear activity monitors. The most common sensors are accelerometers, which measure displacement of the device as well as changes in orientation relative to gravity [14]. For example, by attaching an accelerometer to a shoe, one can estimate the amount of time running and walking based on the presence of periodic motion. To recognize specific activities, there have been many studies placing accelerometers at specific locations on the body - including the head, chest, arm, foot, and thigh, reviewed in [15]. Consistent placement of sensors allows for more consistent signals across individuals. However, the need for consistent placement usually requires clinical supervision. Also, even though accelerometers are inexpensive, they are often part of a dedicated device that needs to be bought and carried. The added cost and inconvenience can make even simple monitors impractical for large-scale, long-term use.

Modern mobile phones have built-in accelerometers that can be used to track movements without the need for an additional device [16], [17]. The collected data can be used for activity recognition [18]–[20] as well as fall detection [21]–[23]. Mobile phones are convenient to use as they have their own power sources, memory storage capabilities, and can transmit data wirelessly. In a phone-based scenario, individuals can simply download an app onto their mobile phone enabling data collection and analysis. Mobile phones allow automatic, convenient, real-time monitoring and recording, which can be invaluable to large-scale studies and personal health monitoring.

In this paper, our goal is to provide evidence that accelerometry using mobile phones can be used to objectively quantify the activity levels of individuals with lower-limb prostheses. We asked participants with prostheses as well as able-bodied participants to carry mobile phones for one week to record their daily activity level. From this data we extracted the amount of movement of participants during that time. Later we compare and correlate these everyday movements to the K-level designation that was assigned clinically.

## Methods

### Participants

Ten participants with transfemoral amputations (5F/5M, ages 53.1±11.9) and 8 control participants (5F/3M, ages 27.2±3.4) were recruited for this study. For the participants with transfemoral amputations, the average height was 168±7 cm and weight was 83±19 kg, resulting in an average BMI of 30±8. There were 7 K3 level participants and one participant in each of the three other levels - K1, K2, and K4. More details on each participant are available in Table 2. All participants were instructed to carry mobile phones for one week to record their everyday activity. During this time participants wore a belt that held a phone in the center of the back. Written, informed consent was obtained for all participants. The Northwestern University institutional review board specifically approved this study.

**Table 2:** Descriptions of individuals with transfemoral amputations.

K-level	Age(yrs)	Sex	BMI30±8	Height(cm)	Weight(kg)	Prosthetic knee,manufacturer	Prosthesis functional capability	Cause	Year sinceamputation
1	64	F	26.6	160	68	3R60, Ottobock	Variable cadence, swing control	Trauma	44
2	62	F	38.4	163	102	3R22, Ottobock	Single cadence	Vascular	24
3_1_	51	F	49.7	157	123	3R60, Ottobock	Variable cadence, swing control	Vascular	12
3_2_	55	M	27.3	178	86	SNS Hydrolic, Mauch	Variable cadence, stance flexion	Trauma	43
3_3_	58	M	20.7	173	62	3R60, Ottobock	Variable cadence, swing control	Trauma	5
3_4_	49	M	32.7	173	98	Black Box, Mauch	Variable cadence, stance flexion	Trauma	14
3_5_	50	F	30.2	163	80	C-leg, Ottobock	Variable cadence, stance flexion,natural walking, stumble recovery	Trauma	18
3_6_	64	M	27.2	175	83	C-leg, Ottobock	Variable cadence, stance flexion,natural walking, stumble recovery	Trauma	33
3_7_	57	M	25.5	173	76	3R60, Ottobock	Variable cadence, swing control	Trauma	6
4	21	F	21.3	160	54	C-leg, Ottobock	Variable cadence, stance flexion,natural walking, stumble recovery	Cancer	7

### Data Acquisition

**Figure 1:**
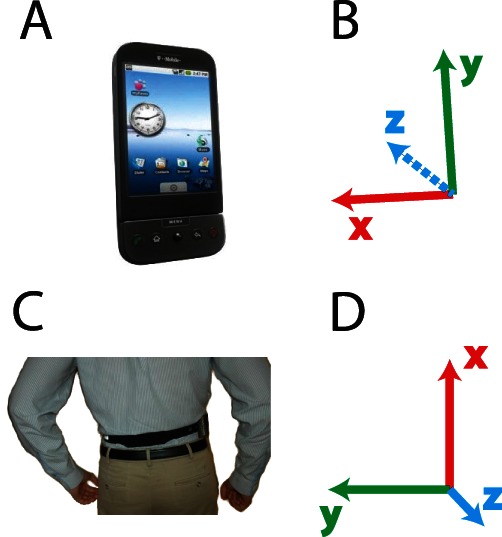
Data acquisition setup. A) The G1 android mobile phone used in this experiment. B) The axes of the tri-axial accelerometer relative to the image in A–xyz as red, green, blue, respectively. C) The phone was placed on the back of the subject so that the three axes pointed up, left, and to the back of the subject, as indicated in D.

The phones were T-mobile G1 phones running Android OS version 1.6. The sampling rate was variable between 15 and 25 Hz, with the higher sampling rate occurring at times of changing acceleration [17]. The phone was positioned such that the accelerometer axes aligned with ‘x’ as vertical (up), ‘y’ as medio-lateral (left), and ‘z’ as antero-posterior (behind) (fig. 1).

### Data Processing

General activity levels for a given day were derived directly from movement as measured by the accelerometer. Clips of these accelerations were classified by the average rate of change of the accelerations of the movement, used as an operational definition of vigor. The percentage of time participants spent at each activity level was used to compare across individuals.

The 3-axis phone accelerometer values were first linearly interpolated to match 20 Hz. All analyses were then performed on 10 second clips. For each axis, the standard deviation of the acceleration values for that clip was computed; the clip was summarized by the mean of all three axes. Table 3 shows the thresholds used in classifying the level of activity of the participant at that time. These thresholds were chosen as they approximately correspond to the labels given and boundaries are clear to communicate. For the purposes of this study, these thresholds were chosen arbitrarily, not for their strict adherence to intuitive concepts of low/medium/high activity. Most importantly, the thresholds are fixed, and the higher the number, the more active the participant.

**Table 3:** Activity level boundaries.

Activity Level	Range (std dev of acc in m/s^2^)
Inactive	0–0.1
Low Activity	0.1–0.5
Medium Activity	0.5–1.0
High Activity	1.0+

Two components of accelerations, the acceleration of the subject as well as gravity, affect accelerometry signals. Therefore, changes in acceleration can come from translational displacements of the phone as well as changes in the orientation of the phone relative to gravity. However, both of these require physical effort. Rotations and translations of the trunk are strenuous and both affect our measure.

The analysis method also had to accommodate times during the week-long data collection when the participants were not wearing their devices. In order to remove the impact of recordings during charging or when the belt was off, we first tried removing samples taken when the phone was still and horizontal. If the orientation of the gravitational vector was within 15 degrees perpendicular to the screen, and the change in acceleration was below 0.1 m/s^2^ standard deviation, the clip was discarded from analysis. However, we found this to be an imperfect approach to determine when someone was not wearing the device; for example, this approach could incorrectly classify long periods of lying down flat on the front or back as “not worn”, leading to further errors. Since we were uncertain specifically when someone was not wearing the device, we found it was better to measure the relative amount of activity at different levels (e.g. percent of movement that was highly activity) rather than estimate the total amount of time (hours highly active). For this reason, in the results presented we only used clips when the change in acceleration was above 0.1 m/s^2^. Although we collected inactive data, the analysis reflects only the times when the subjects are active.

Summaries of activity over participant weeks were performed by totaling the amount of time spent in each level of activity over the entire week. In order to give approximate confidence intervals, we used bootstrapping to simulate variations in activity of participants based on the limited data that was recorded. We first determined the total time in each activity level for each day. Randomly and with replacement we selected seven days to generate one bootstrap simulated week, and totaled the time in each activity level for each bootstrap sample. After 1000 random samples were selected, the 2.5% and 97.5% samples were selected as the bounds for the 95% confidence interval. For all comparisons between groups, all analyses were performed with one-tailed sign-rank tests unless otherwise specified.

## Results

Both able-bodied controls and individuals with transfemoral amputations were instructed to carry the phones for one full week. The phones were worn on belts (fig. 1) and continuously measured accelerations. This setup allowed us to continuously monitor participant movements during everyday life.

**Figure 2:**
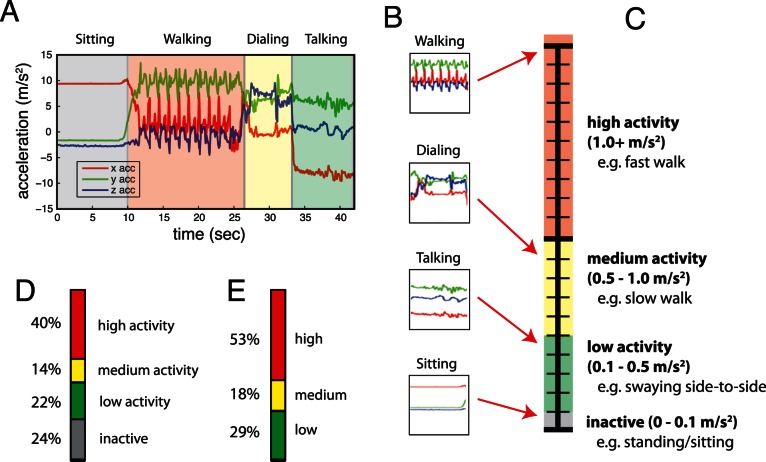
Schematic of data analysis. A) Example data acquired from normal cell phone use, recorded for this illustration. B) 10 second segments extracted from part A. The labels are only used for interpretation. C) The clips were then placed on a scale by their averaged standard deviations of the accelerations for each axis and binned appropriately. Colors are associated with each bin of activity. Example activities are given for each bin when the phone is worn on the belt. D) Proportions in those bins when including inactive data. E) Proportions when excluding inactive data–used to exclude all times when the phone is not worn or the subject is not moving.

The week-long accelerometer recordings are distilled into a general measure of activity for each participant (fig. 2). Different activities led to distinct acceleration patterns. These patterns were scored based on the measured movement of the device (see methods). The amount of movement, as measured by changes in acceleration on the phone, is indicative of the types of activities participants are engaged in. We observe the general amount of activity by observing the fraction of time spent at each of these levels of activity.

**Figure 3:**
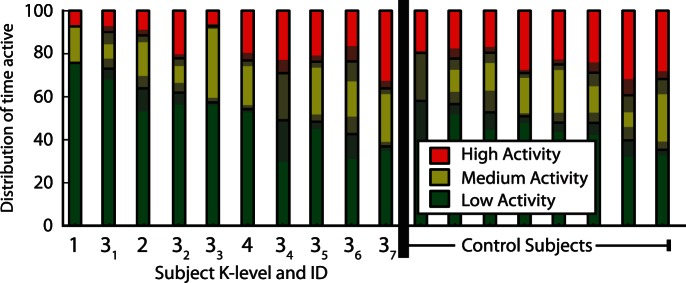
The distribution of activity level for each subject. To aid interpretation, the participants have been ordered based on overall activity level (medium+high). The IDs correspond to the subject K-levels, and subscripts are given to match the description of subjects in Table 2. The gray transparency indicates the 95% confidence interval using bootstrapping over days recorded.

To analyze the relationship between K-level and activity, we observe the fraction of time spent at the combined medium and high levels of activity for all participants (fig. 3). There was a tendency that participants with amputations had lower levels of activity than the 8 able-bodied participants (p = 0.08, one-tailed rank sums). More specifically, the K1 and K2 subjects are less active than any of the control subjects. Moreover, both K1 and K2 and two of the K3 subject showed less high-level activity than any of the healthy controls. Despite high inter-individual variations, even this small scale study showed trends that K levels co-vary with high level activity.

Importantly, our analysis allows an understanding of the precision of the activity levels. Using bootstrapping across days we calculated how precise the estimates of activity levels are (Fig. 3, errorbars in gray). We find that across days our technique yields similar estimates of activity levels. The 95% confidence errors are quite small (mean interval = 10.1% ±2.1 std. err, median interval = 6.7%). Much of the day-to-day variability was driven by a small number of participants that only carried the phone for a short period of time on certain days. This is evident in the difference between the mean and median interval due to the high degree of skew. If worn consistently, this technique has good test-retest reliability.

There is a wide variation in the activity of the participants with amputations; some individuals show even more high-activity than able-bodied controls. Here, we consider a few potential sources of these variations. Among the seven K3 participants we observe a weak relationship between the BMI of participants and their level of activity (r = −0.62, p = 0.07 one-tailed), indicating a potential confound of participant weight. We also tested the effect of different prosthetic legs–comparing the five legs with fewer features (3R22, 3R60) to the five with more features (Mauch, C-leg), but no statistically significant relationship was found (p = 0.11, rank sums). Although analyses did not present definitive causes for the variations among amputee activity, possible trends are indicated that could be considered in later studies.

## Discussion

In this study we analyzed the relationship between the activity level of participants when using mobile phones and their designated K-level. Given a larger sample size, an estimated range of K-level should be possible from data conveniently measured using a mobile phone. Unlike typical clinical tests, this data represents how a person actually moves in their day-to-day life, and is thus closer to how they would move outside the clinical setting than traditional clinically-scored measures. Such an evaluative tool for justifying a K-level designation can provide support for clinical decisions that currently have little quantitative support.

Currently there are metrics that can be used to estimate K-level. There are a number of self-report questionnaires a physician can use to gauge a patient’s current ability or desire to ambulate [24]–[28], but as with all self-report questionnaires, they are subject to bias, especially with regard to assessing the level of activity they would like to reach. Perhaps a more accurate assessment is to combine the ease of a survey format, but have the judgments and scoring be performed by a clinician as is done with the Barthel Index [29], the Functional Independence Measure [30], and the Amputee Mobility Predictor [31]. However, to avoid the need for clinical judgment during scoring, there are a number of physical performance metrics which can be used to establish a patient’s current ability–e.g. the six minute walk test [32], timed up and go [33], and berg balance [34] to name a few. Importantly, these questionnaires, surveys, and physical measures are measuring patients as they are presented in the clinic, and using their self-assessments to determine their future functional level. These also do not provide a metric by which one could assign a patient to any of the functional K-levels.

Although this study applies mobile phones to track movement of subjects with prosthetic legs, a number of other approaches have used direct kinetic measurements of this population to characterize their capacity to move. This can include measuring the forces and moments in prosthetic limbs [35]–[38], with the goal of determining functional outcomes [39]. Direct force and kinetic measurements on the prosthetic limb can be used to characterize specific activities, such as walking [40], [41] or incidents of falls [42], [43]. Movement and force data can be collected from prostheses and related to daily activities [44]–[47]. There is previous work that estimated function levels directly from at-home monitoring. For example, Orthocare Innovations uses an ankle-worn device, the StepWatch [48], [49], to record steps over the course of a week. By observing the person’s stepping patterns using this device, and performing an analysis using their proprietary Galileo clinical outcomes assessment method, they produce an estimated K-level, with a fractional precision to indicate a relative high or low functional ability within a K-level category. We believe this approach, using a dedicated device and analysis tools based on the device output, is promising. However, the price of each StepWatch device is currently over $500 and the proprietary analysis tools add more to the cost, which when compared to the typical cost of a mobile phone is substantially more. Moreover, full accelerometry should be able to provide more detailed information about patient activities during everyday life [50]–[52]. There are a number of ways to directly measure movements and forces on the prosthesis, or have participants wear dedicated devices elsewhere, and these devices can also provide information used to estimate function outcomes.

Our work uniquely demonstrates that it is possible to use mobile phones to measure the amount of daily activity in individuals with lower-limb amputations. We observe a relationship between the amount of daily activity and functional level, which suggests that future studies could potentially use this information for K-level prediction. The current reliance on clinical measurements and self-reported abilities may not reflect the actual at-home use of prosthetic devices, which can lead to both over and under-prescribing. Accurate prosthetic prescriptions are important to further reign-in health care costs and avoid undue adjustments for the expected growth of lower-limb amputations. By incorporating objective, convenient, and inexpensively-acquired data on the actual use of lower-limb prostheses, clinicians will have more information at their disposal to make an accurate, cost-effective, and functionally appropriate prescription for people with lower-limb amputations.
